# Gene Expression Profile of High IFN-γ Producers Stimulated with *Leishmania braziliensis* Identifies Genes Associated with Cutaneous Leishmaniasis

**DOI:** 10.1371/journal.pntd.0005116

**Published:** 2016-11-21

**Authors:** Marcia W. Carneiro, Kiyoshi F. Fukutani, Bruno B. Andrade, Rebecca P. Curvelo, Juqueline R. Cristal, Augusto M. Carvalho, Aldina Barral, Johan Van Weyenbergh, Manoel Barral-Netto, Camila I. de Oliveira

**Affiliations:** 1 Instituto Gonçalo Moniz, Fundação Oswaldo Cruz (FIOCRUZ), Salvador, Brazil; 2 Multinational Organization Network Sponsoring Translational and Epidemiological Research (MONSTER) Initiative, Fundação José Silveira, Salvador, Brazil; 3 Department of Microbiology and Immunology, Rega Institute for Medical Research, KU Leuven, Leuven, Belgium; 4 Federal University of Bahia School of Medicine, Salvador, Brazil; University of Notre Dame, UNITED STATES

## Abstract

**Background:**

The initial response to *Leishmania* parasites is essential in determining disease development or resistance. *In vitro*, a divergent response to *Leishmania*, characterized by high or low IFN-γ production has been described as a potential tool to predict both vaccine response and disease susceptibility *in vivo*.

**Methods and findings:**

We identified uninfected and healthy individuals that were shown to be either high- or low IFN-γ producers (HPs and LPs, respectively) following stimulation of peripheral blood cells with *Leishmania braziliensis*. Following stimulation, RNA was processed for gene expression analysis using immune gene arrays. Both HPs and LPs were shown to upregulate the expression of *CXCL10*, *IFI27*, *IL6* and *LTA*. Genes expressed in HPs only (*CCL7*, *IL8*, *IFI44L* and *IL1B)* were associated with pathways related to IL17 and TREM 1 signaling. In LPs, uniquely expressed genes (for example *IL9*, *IFI44*, *IFIT1* and *IL2RA*) were associated with pathways related to pattern recognition receptors and interferon signaling. We then investigated whether the unique gene expression profiles described here could be recapitulated in vivo, in individuals with active Cutaneous Leishmaniasis or with subclinical infection. Indeed, using a set of six genes (*TLR2*, *JAK2*, *IFI27*, *IFIT1*, *IRF1* and *IL6*) modulated in HPs and LPs, we could successfully discriminate these two clinical groups. Finally, we demonstrate that these six genes are significantly overexpressed in CL lesions.

**Conclusion:**

Upon interrogation of the peripheral response of naive individuals with diverging IFN-γ production to *L*. *braziliensis*, we identified differences in the innate response to the parasite that are recapitulated *in vivo* and that discriminate CL patients from individuals presenting a subclinical infection.

## Author Summary

Control and development of Cutaneous Leishmaniasis (CL) are dependent on the host immunological response. One of the key molecules in determining elimination of *Leishmania* parasites from the infected host cell is the cytokine interferon gamma (IFN-γ). The aim of this study was to investigate which immune response genes are associated with the production of IFN-γ in the context of *Leishmania* infection. We identified individuals that are high- or low IFN-γ producers upon stimulation of their peripheral blood cells with *Leishmania* parasites. We then determined the immune gene expression profile of these individuals and we identified a set of genes that are differentially expressed comparing high- and low IFN-γ producers. The expression of these genes was also evaluated in patients with CL and in individuals with a subclinical *Leishmania* infection (SC). In this setting, the overall pattern of expression of this particular gene combination discriminated the CL patients x from SC individuals. Understanding the initial response to *Leishmania* may lead to the identification of markers that are associated with development of CL.

## Introduction

Cutaneous Leishmaniasis (CL) caused by *Leishmania braziliensis* is characterized by a broad spectrum of clinical manifestations, ranging from localized CL to Mucosal Leishmaniasis (rev. in [1]). A hallmark feature of the immunological response in localized CL is a strong Th1 type immune response to soluble *Leishmania* antigen (SLA), demonstrated by a positive delayed type hypersensitivity (DTH) reaction to the *Leishmania* skin test, as well as lymphocyte proliferation and high levels of IFN-γ and TNF-α [2–4].

Since T-cell-mediated immunity plays a central role in the host’s response to intracellular parasites, *in vitro* experimental settings have been used to address the initial lymphocyte response to *Leishmania*: PBMCs from naive volunteers stimulated with *L*. *major* produce mainly IFN-γ and this effect is regulated by IL-10 and IL-12 [5]. Using an *in vitro* priming system with *Leishmania amazonensis* antigen, Pompeu et al. showed that cells from naïve individuals produce either high or low amounts of IFN-γ [6]. These two patterns of *in vitro* anti-*Leishmania* response correlated with the *in vivo* post-vaccination response: low *in vitro* IFN-γ producers exhibit a delayed response to vaccination with SLA, whereas an accelerated immune reaction vaccine is observed in those who were high IFN-γ producers [6]. Upon stimulation with *L*. *amazonensis*, high IFN-γ producers also secrete more TNF [6], more IL-12 and less IL-13 [7]. These results indicate that a low IFN-γ response *in vitro* accompanies a slower IFN-γ production *in vivo* and authors suggested that *in vitro* responses could be used to predict, for example, the pace of post vaccination responses.

IFN-γ, produced primarily by T cells and natural killer cells, is an important mediator of macrophage activation and intracellular pathogen killing, including *Leishmania*. We previously demonstrated that PBMCs from healthy uninfected individuals respond differently to *Leishmania* stimulation (secreting either high or low amounts of IFN-γ). In this study, we aimed at characterizing the immune gene signature that parallels these two responses. Further, we investigated whether such *in vitro* responses had *in vivo* equivalents by probing the gene expression of CL patients and that of individuals presenting a subclinical infection which is associated with absence of lesions, a positive *Leishmania* skin test (LST) [8], and lower levels of both IFN-γ and TNF [9]. We expand the current knowledge in the field by identifying genes that are expressed in association with the capacity to produce IFN-γ upon stimulation with *Leishmania braziliensis*. The immune signature associated with IFN-γ production also discriminates CL patients from individuals with subclinical infection.

## Methods

### Study population and ethics statement

Peripheral Blood Mononuclear Cells (PBMCs) were obtained from healthy uninfected individuals (*n* = 9) recruited in the city of Salvador (Bahia state, Brazil), where *L*. *braziliensis* transmission in not endemic (S1 Table). These individuals had negative serology results for leishmaniasis, negative serology for Chagas’ disease, hepatitis and human immunodeficiency virus. CL patients and individuals presenting a subclinical (SC) infection were recruited from the area of Jiquiriça (Bahia state, Brazil), where *L*. *braziliensis* transmission is endemic (S2 Table). Patients with active CL (*n* = 5) were diagnosed based on the presence of a typical clinical leishmaniasis lesion, a positive *Leishmania* skin test (LST) and documentation of parasites in culture or by histopathology. SC individuals (*n* = 8) were identified in the same endemic area, following a medical interview. These individuals had no history of past CL (absence of scars consistent with CL or Mucosal Leishmaniasis in the skin, nose and soft palate) and a positive LST to *Leishmania*. This research was conducted with the approval of the ethical committee of Centro de Pesquisas Gonçalo Moniz (CPqGM), Fundação Oswaldo Cruz (FIOCRUZ) (Salvador, Bahia, Brazil; 177/2008) and Comissão Nacional de Ética em Pesquisa (Brazilian National Ethics Committee, Brazil), and written informed consent was obtained from each participant.No minors participated in the study.

### Parasite culture

*L*. *braziliensis* promastigotes (strain MHOM/BR/01/BA788) were grown in Schneider medium (Sigma), supplemented with 100 U/ml of penicillin, 100 ug/ml of streptomycin and 10% heat-inactivated fetal calf serum (all from Invitrogen).

### Cell culture, stimulation and IFN-γ detection

PBMCs from healthy individuals (*n* = 9) were obtained from heparinized venous blood layered over a Ficoll-Hypaque gradient (GE Healthcare). Cells were washed and resuspended in RPMI1640 supplemented with 10% human AB serum, 100 IU/ml of penicillin and 100μg/ml of streptomycin (all from Invitrogen). PBMCs (3x10^6^/ml) were placed in the wells of a 24-well plate at 500 μl per well. *L*. *braziliensis* stationary phase promastigotes were added to the cultures at a parasite/cell ratio 1:1. Control cultures were maintained in medium only. Cultures were performed in triplicate and maintained at 37°C/5% CO_2_. After 72h, IFN-γ levels in culture supernatants were determined by ELISA (R&D Systems), following manufacturer´s instructions.

### Quantitative Real-Time PCR

PBMCs obtained from previously defined HPs (*n* = 3) and LPs (*n* = 3) were stimulated with *L*. *braziliensis* promastigotes for 72h, as described above. After stimulation, total RNA was obtained using Trizol (Invitrogen), according to manufacturer's instructions. RNA (500 ng) was suspended in 50μl DEPC-treated water and stored at –70°C until use. cDNA was synthesized from DNAse-treated RNA by reverse transcription using RT^2^ First Strand kit (Qiagen), following manufacturer´s instructions. cDNA obtained from cultures stimulated with *L*. *braziliensis* or from control cultures (maintained in the absence of stimulus) was then employed in PCR array analysis using RT^2^ Real-Time SYBR Green PCR Master Mix (Qiagen) and the following human RT^2^ Profiler PCR arrays: Th1 & Th2 responses, Toll-Like Receptor Signaling Pathway, Interferon & receptors and Chemokines & Receptors (Qiagen), following manufacturer´s instructions. Reactions were performed on ABI 7500 Sequence Analyzer (Applied Biosystems). Fold changes in gene expression between *L*. *braziliensis*-stimulated and control cultures were calculated using the RT^2^ Profiler PCR array data analysis tool, based on the ΔΔCt method, after normalization to housekeeping genes, determined by the manufacturer. A gene was considered differentially expressed when fold change was above or below 2, compared to control cultures, and *p*<0.05 when comparing the different groups [HPs (*n* = 3) and LPs (*n* = 3) (such genes are indicated in Supplemental Data Set 1)]. For confirmation of results obtained in the RT^2^ Profiler PCR arrays, PBMCs from HPs (*n* = 3) and from LPs (*n* = 3) were stimulated with *L*. *braziliensis* or were cultured in the absence of stimulus (control). RNA was employed in individual quantitative Real Time PCR (qRT-PCR) reactions using primers for IFNG, CXCL10, IFI27, IL6 and IRF1 designed using Primer Express Software (ThermoFisher Scientific), reactions were performed as described elsewhere [10]. qRT- PCRs reactions were run in triplicates for each gene of interest and compared with a housekeeping gene (*GAPDH*), also using the ΔΔCt method [11]. We compared the stability of B2M, HPRT1, ACTB and GAPDH (housekeeping genes) in our PBMC samples (stimulated or not with *L*. *braziliensis*). Only ACTB and GAPDH displayed normal distribution (by both Shapiro-Wilk and Kolmogorov-Smirnov tests) and low Coefficient of Variation (B2M 94.39%, HPRT1 277.36%, GAPDH 60.61% and ACTB 50.75%). However, GAPDH displayed lowest skewness (0.03 vs. 0.65 for B2M, 3.97 for HPRT1 and 0.28 for ACTB), i.e. was very close to Gaussian distribution across all samples. Also in this experiment, IFNG transcripts were strongly correlated to B2M (r = 065, p = 0.004), HPRT1 (r = -0.44, p = 0.078) and ACTB (r = -0.44, p = 0.080) transcripts, in agreement with their annotation as IFN-regulated genes. In contrast, GAPDH transcripts were not significantly correlated to *IFNG* transcripts (r = -0.13, p = 0.62). To eliminate this small, but possible bias, we choose GAPDH as the housekeeping gene for the validation experiments. As a positive control, PBMCs from HPs and LPs were stimulated with Phytohaemagglutinin (SIGMA) (10μg/ml); RNA was extracted and submitted to qRT-PCR for IFNG expression as described above. PBMCs from CL patients (*n* = 5) and from SC individuals (*n* = 8) were also stimulated with *L*. *braziliensis*; RNA was then submitted to qRT-PCR against IFI27, IFIT1, TLR2, IRF1, JAK2 and IL6 using custom designed primers. In these experiments, PBMCs from CL patients and SC individuals were obtained before placement of LST.

### Data analysis

IFN-γ levels in culture supernatants and gene expression levels after PBMC stimulation were compared by the Mann-Whitney test using Prism (GraphPad, V. 6.0). Functional analyses were generated using Ingenuity Pathway Analysis (IPA, QIAGEN, V. 01–06), using as the Reference Set (Population of genes considered for p-value calculation), a User Data Set instead of the Ingenuity Knowledge Base. The User Data Set consisted of the 269 genes evaluated in the arrays. The genes differentially modulated in HPs (32) and LPs (29) were tested against this panel of 269 genes collectively present in the arrays. Hierarchical cluster analysis using the Ward’s method with bootstrap and principal component analysis (PCA) were performed using and JMP Statistical Discovery (V 12). The number of transcripts for (*IFI27*, *IFIT1*, *TLR2*, *IRF1*, *JAK2* and *IL6)* was quantified in data sets GSM1341365 [12] and GSM1560512 [13] containing transcriptomic data from CL lesions and from healthy (control skin). Data were normalized using RMA (Robust Multichip Average) for each dataset and the number of transcripts were compared by unpaired t-test using Prism (GraphPad Software, V 6.0). For all comparisons, a *p*-value ≤ 0.05 was considered significant.

## Results

### Defining high and low producers based on IFN-γ production after stimulation with *L*. *braziliensis*

**Fig 1:**
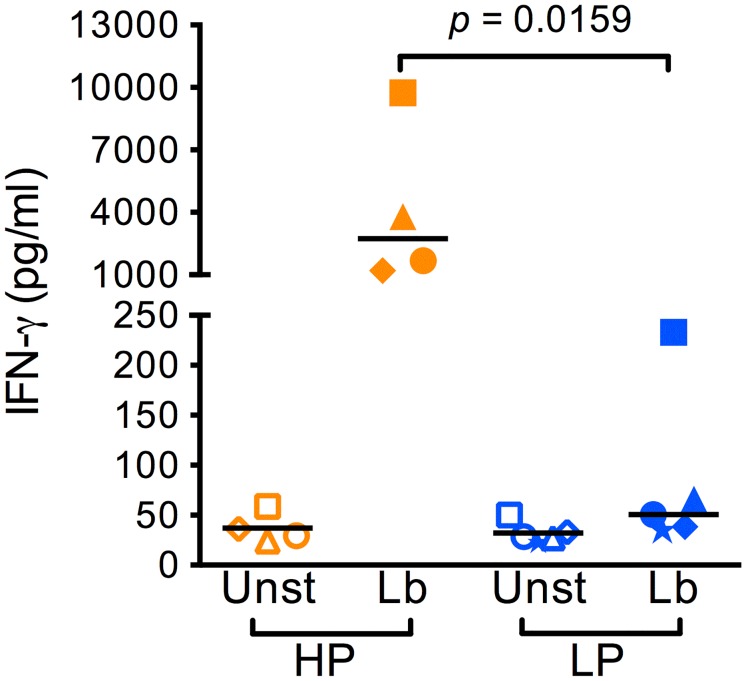
Naïve individuals are High- or Low- IFN-γ producers upon stimulation with *Leishmania* braziliensis promastigotes. PBMCs were cultured with *L*. *braziliensis* for 72h (Lb) or left unstimulated (Unst). Supernatants were collected and IFN-γ levels were determined by ELISA. Data from one representative experiment are shown individually for High Producers (HP) (*n* = 4) and Low Producers (LP) (*n* = 5). Samples from three HP (orange) and three LP (blue) individuals, depicted as triangle, diamond and circle were used in subsequent experiments.

We have previously shown that PBMCs from naive volunteers exposed to *Leishmania* secrete IFN-γ in high or low amounts, allowing the classification of such individuals as either high- or low- producers (HPs and LPs, respectively) [6]. Herein, we aimed at further dissecting these differential responses to *Leishmania*, particularly in relation to the expression of immune-related genes that parallels IFN-γ production following exposure to *L*. *braziliensis*. Initially, PBMCs from healthy individuals were exposed to *Leishmania* promastigotes and supernatants collected after 72h were assayed for the presence of IFN-γ. The IFN-γ concentrations detected in culture supernatants evidenced individuals who are high IFN-γ producers (HPs) and individuals who are low IFN-γ producers (LPs) (Fig 1). Among all volunteers (*n* = 9), the median IFN-γ level was 233 pg/ml, this value was further considered as the cut off for defining HPs and LPs. Therefore, in HPs, IFN-γ levels in culture supernatants were >300pg/ml whereas LPs were defined as presenting IFN-γ levels <300pg/ml (Fig 1). As a positive control, PBMCs from HPs and LPs were stimulated with a mitogen and IFNG expression, as accessed by qRT-PCR, did not differ significantly between the two groups (median IFNG expression was 11.24 for HPs and 7.66 for LPs). The difference in IFN-γ levels observed in HPs and LPs did not result from distinct responses to *L*. *braziliensis* infection since both HPs and LPs displayed similar percentages of infected macrophages and of amastigotes per infected macrophages (S1 Fig). Also, the significant difference in IFN-γ production comparing HPs and LPs was replicated in a subsequent experiment (S2 Fig).

### Gene expression profiling of High IFN-γ Producers vs. Low IFN-γ Producers

**Fig 2:**
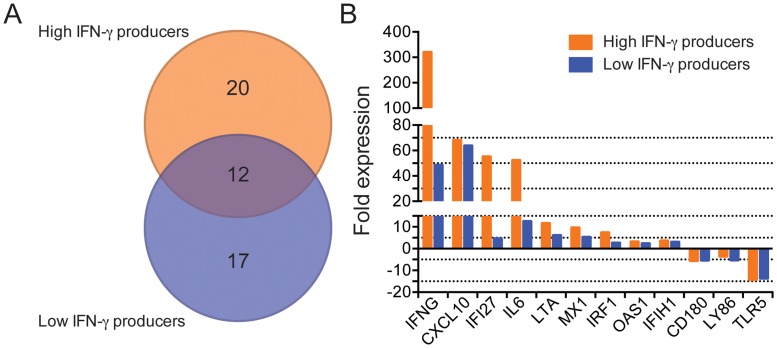
Genes modulated in High- or Low- IFN-γ producers following stimulation with *L*. *braziliensis*. PBMCs from High IFN-γ Producers (HPs) (*n* = 3) and from Low IFN-γ Producers (LPs) (*n* = 3) were stimulated with *L*. *braziliensis* for 72h. Gene expression was determined using total RNA and arrays covering Th1-Th2-Th3 responses, IFN-γ and receptors, chemokines and receptors and TLRs. (A) Venn diagram depicting the total number of genes differentially modulated in HPs only, LPs only or modulated in both categories, as evidenced by arrays analysis (see materials and methods). (B) Fold expression of the 12 genes differentially modulated in both HPs and LPs, as evidenced by array analysis (see materials and methods).

To investigate the expression profile of immune genes paralleling the two patterns of *L*. *braziliensis*-induced IFN-γ responsiveness (HPs vs. LPs), total RNA obtained from *L*. *braziliensis*-stimulated cultures was employed in PCR arrays covering Th1-Th2-Th3 responses, IFN and receptors, chemokines and TLRs. Among the 269 genes evaluated, we identified 49 genes differentially expressed in *L*. *braziliensis*-stimulated cultures relative to control cultures, considering both HPs and LPs (fold change above or below 2, compared to control cultures, and *p*<0.05). These genes (indicated in Supplemental Data Set 1) were identified based on differential expression following stimulation with *L*. *braziliensis*, as described in Materials and Methods. Twenty genes were uniquely modulated in HPs whereas 17 genes were uniquely modulated in LPs (Fig 2A). Twelve genes were commonly modulated in both HPs and LPs and, within these, *IFNG* was the top up-regulated gene (Fig 2B). *IFNG* expression was ~8-fold higher in HPs compared to LPs, a finding that recapitulated the higher IFN-γ levels detected in culture supernatants (Fig 1). In addition to *IFNG*, eleven other genes were also modulated in both HPs and LPs (Fig 2B) including *CXCL10* and *IL6* (for which the expression level was ~4-fold higher in HPs compared to LPs). HPs also upregulated genes associated with a type I interferon (IFN) response such as *OAS1*, *MX1* and *IRF1*. *IFI17*, upregulated in response to stimulation by interferon [14], was highly expressed in HPs. On the converse, expression of *CD180*, *LY86* and *TLR5* was suppressed at similar levels in both HPs and LPs (Fig 2B). These results demonstrate that HPs and LPs modulated, in general, a similar number of genes, twelve of which were common between both categories.

**Fig 3:**
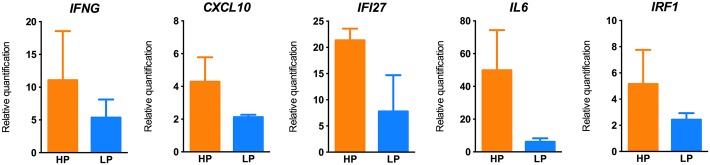
qRT-PCR validation of genes commonly modulated in High- and Low- IFN-γ producers. PBMCs from High Producers (HPs) (*n* = 3) and Low Producers (LPs) (*n* = 3) were stimulated with *L*. *braziliensis* for 72h. Relative expression of *IFNG*, *CXCL10*, *IFI27*, *IL6* and *IRF1* was evaluated by qRT-PCR. Gene expression is represented as fold change of stimulated over unstimulated cultures, normalized to a housekeeping gene. Bars represent the mean ± SEM.

We then selected the top four genes modulated in PCR arrays in both HPs and LPs (IFNG, CXCL10, IFI27 and IL6) plus IRF1 and validated their expression by qRT-PCR, using custom designed primers (Fig 3). Reactions performed with RNA from the same HPs (*n* = 3) and LPs (*n* = 3) confirmed that HPs express higher levels of *IFNG*, *CXCL10*, *IFI27*, *IL6* and *IRF1* compared to LPs. Therefore, higher production of IFN-γ in response to *L*. *braziliensis* stimulation is accompanied by upregulation of a series of known IFN-stimulated genes such as CXCL10, IFI27 and IRF1.

**Fig 4:**
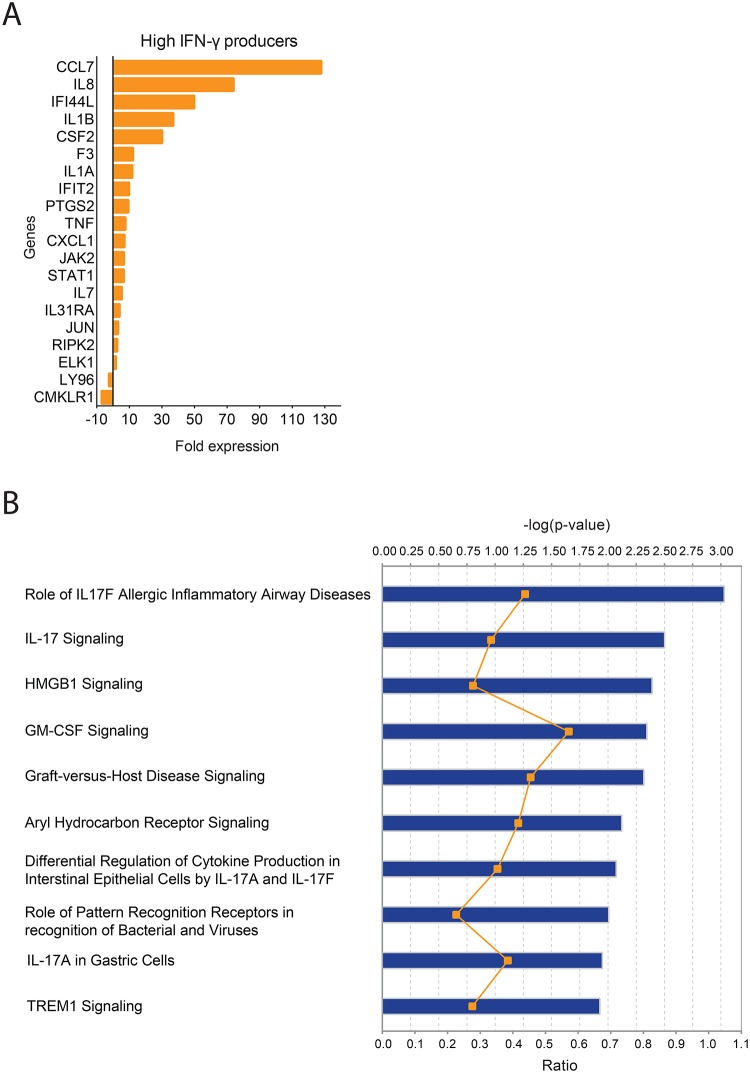
Expression profile of genes uniquely modulated in High- IFN-γ producers. (A) PBMCs from High IFN-γ Producers (HPs) (*n* = 3) were stimulated with *L*. *braziliensis* for 72h. Gene expression was determined using total RNA and immune gene arrays. Fold expression of the genes modulated in HPs or in LPs only, as evidenced by PCR array (see materials and methods). (B) Top canonical pathways identified in HPs by Ingenuity Pathway Analysis. Levels of significance are given by the right-tailed Fisher exact test. The negative log *P* value (blue bars), along the x-axis, increases as a pathway is more significantly associated with the set of genes expressed in HPs. The ratio (orange line) indicates the proportion of upregulated genes relative to all the genes present in a pathway.

Within the genes differentially expressed only in HPs (*n* = 20; indicated with p< 0.05 in (Supplemental Data Set 1), *CCL7*, *IL8*, *IFI44L*, *IL1B* and *CSF2* were expressed >20-fold in *L*. *braziliensis*-stimulated cultures compared to control cultures (Fig 4A). Genes coding for chemokines (*CXCL1)*, cytokines *(IL1A*, *TNF*, *IL7)*, transcription factors *(STAT1*, *ELK1)*, protein kinases *(JAK2*, *RIPK2)*, *F3* (coagulation factor III), *IFIT2*, *IL31RA* and *JUN* were also upregulated in HPs whereas *LY96* and *CMKLR1* were downregulated (Fig 4A). Following the identification of the immune genes differentially expressed in HPs [*n* = 32, 12 common genes (Fig 2) plus 20 unique genes, indicated with p< 0.05 in Supplemental Data Set 1], we inquired the immune pathways associated with that expression profile. Among the pathways significantly enriched were IL17 and TREM1 (triggering receptor expressed on myelois cells) signaling (Fig 4B).

**Fig 5:**
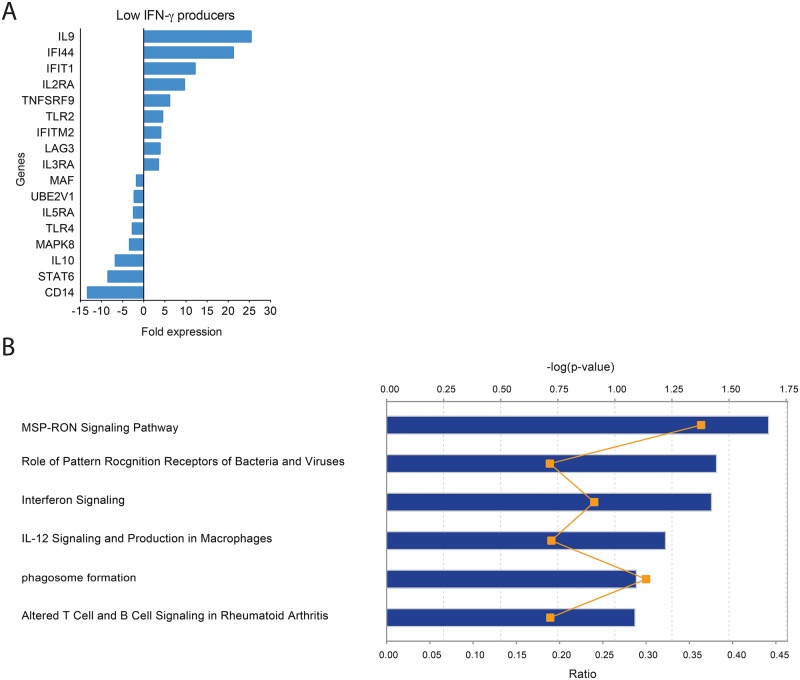
Expression profile of genes uniquely modulated in Low- IFN-γ producers. (A) PBMCs from Low IFN-γ Producers (LPs) (*n* = 3) were stimulated with *L*. *braziliensis* for 72h. Gene expression was determined using total RNA and immune gene arrays. Fold expression of the genes modulated in HPs or in LPs only, as evidenced by PCR array (see materials and methods). (B) Top canonical pathways identified in LPs by Ingenuity Pathway Analysis. Levels of significance are given by the right-tailed Fisher exact test. The negative log *P* value (blue bars), along the x-axis, increases as a pathway is more significantly associated with the set of genes expressed in LPs. The ratio (orange line) indicates the proportion of upregulated genes relative to all the genes present in a pathway.

In LPs, fold expression was in general lower than in HPs and the uniquely upregulated genes (*n* = 17; genes are indicated with p< 0.05 in Supplemental Data Set 1) were cytokines (*IL9*), pattern recognition receptors (*TLR2*), cytokine receptors (*IL2RA*, *TNFSRF9*, *IL3RA*), IFN-related molecules (*IFI44*, *IFIT1*, *IFITM2*) and *LAG3*, which belongs to the Ig superfamily (Fig 5A). Moreover, in LPs, *MAF*, *IL5RA*, *TLR4*, *MAPK8*, *IL10*, *STAT6* and *CD14* expression was suppressed upon stimulation with *L*. *braziliensis*. As before, upon identification of the differentially expressed genes in LPs [*n* = 28, 12 common genes (Fig 2) plus 17 unique genes, indicated with p< 0.05 in Supplemental Data Set 1], pathways involved in the role of pattern recognition receptors, IL-12 and in interferon signaling were enriched (Fig 5B). Altogether, our findings show that HPs, but no LPs, modulate the expression of genes associated with the inflammatory response (*CCL7*, *IL8*, *IL1B*, *IL1A* and *TNF)* and the overall immune signature is associated with IL-17-related pathways.

### Gene expression profile discriminates CL patients vs. individuals with subclinical infection

Following the observation that HPs modulate genes associated with an inflammatory response, we hypothesized that these features could have an *in vivo* equivalent in CL patients. In CL, the immune response is characterized by strong production of IFN-γ and TNF, cytokines that are important for controlling infection but that are also associated with pathogenesis (rev. in [15]). The *in vivo* counterpart to LPs would be individuals with subclinical infection (SC). SC individuals do not present a clinical lesion, display a positive LST [8] and lower IFN-γ and TNF production upon PBMC stimulation [9,16]. We therefore selected genes modulated in both HPs and LPs (IL6, IFI27 and IRF1), modulated in HPs only (JAK2) or in LPs only (IFIT1 and TLR2). Additionally, these genes have been implicated in the control or progression of *Leishmania* infection: type I IFN positively regulates SOD1 levels, decreasing superoxide and increasing *L*. *amazonensis* and *L*. *braziliensis* burden in vitro [17,18]; TLR2 has been associated with reduced pathology in vaccination studies [19]; JAK2 is modulated in *Leishmania*-infected cells [20] and IL6 has been associated with CL/mucosal leishmaniasis susceptibility [21].

**Fig 6:**
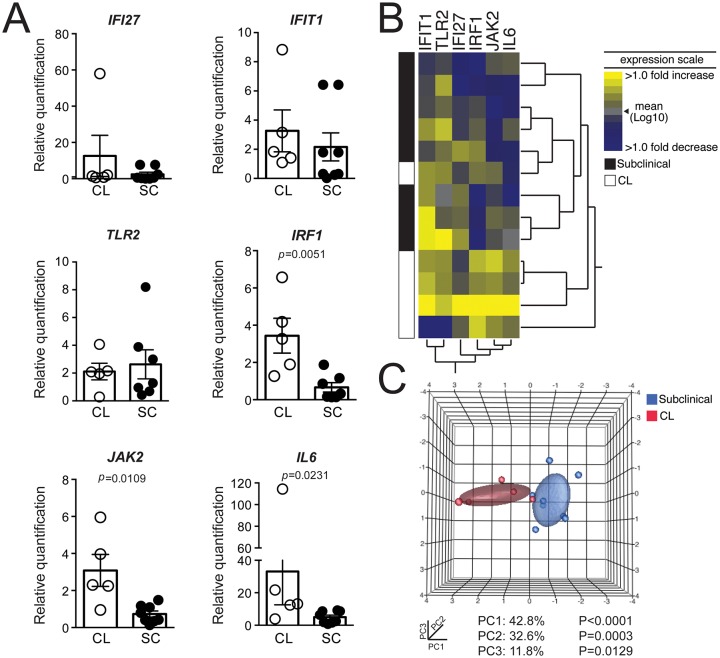
Genes modulated in High- and Low- IFN-γ producers discriminate subclinical *L*. *braziliensis* infection from active CL. (A) PBMCs from CL patients (*n* = 5) and SC individuals (*n* = 8) were stimulated with *L*. *braziliensis* for 72h. Relative expression of *IFI27*, *IFIT1*, *TLR2*, *IRF1*, *JAK2* and *IL6* was evaluated by qRT-PCR. Gene expression is represented as fold change of stimulated over unstimulated cultures, normalized to a housekeeping gene and each symbol represents one individual. (B) A heat map was designed to depict the pattern of gene expression [shown in (A)] of SC individuals) vs. active CL and two-way hierarchical cluster analysis (Ward’s method) of differentially expressed genes was performed. Expression scale for each gene represents the log2-fold change from the mean. (C) Principal component analysis of the differentially expressed genes [depicted in (A)] showing PC1 (x axis), PC2 (y axis) and PC3 (z axis).

**Fig 7:**
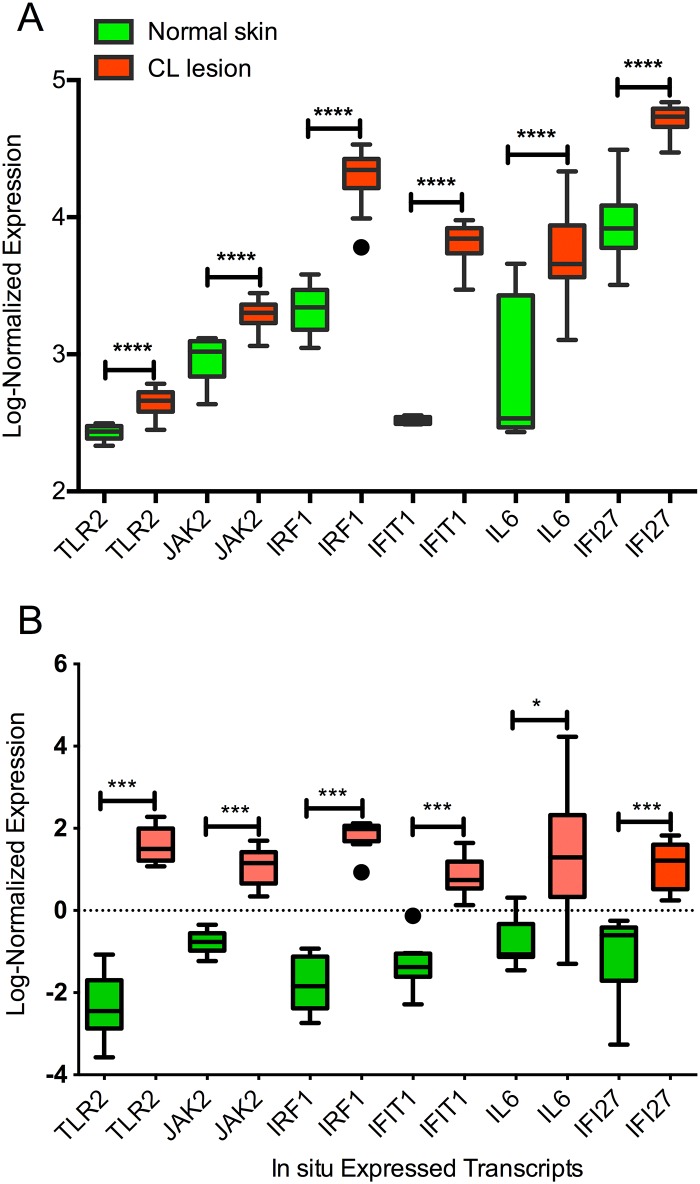
Genes modulated in High- IFN-γ producers are upregulated in CL lesions. The number of transcripts for each gene was quantified in Data sets GSM1341365 [12] and GSM1560512 [13]. Data were normalized using RMA (Robust Multichip Average) for each dataset, log-transformed expression for each gene is shown as box and whiskers (displaying quartile range) and outliers (identified by Tukey’s test). (t-test, ****p<0.0001, ***p<0.001, **p<0.01, *p<0.05).

PBMCs obtained from CL patients and from SC individuals were stimulated with *L*. *braziliensis* and expression of these genes was measured by qRT-PCR. Expression of *IFI27*, *IFIT1* and *TLR2* did not differ significantly comparing CL patients and SC individuals (Fig 6A) but expression of *IRF1*, *JAK2* and *IL6* was significantly higher in CL individuals. Hierarchical clustering showed that the pattern of expression of *IFIT1*, *TLR2*, *IFI27*, *IRF1*, *JAK2* and *IL6* successfully discriminated CL patients from SC individuals (Fig 6B) and this result was further confirmed by principal component analysis (Fig 6C) showing that immune markers that accompany High- or Low IFN-γ production in naïve individuals, following exposure to *L*. *braziliensis*, are recapitulated *in vivo*. To address whether these genes are also expressed in CL lesions, we performed *in silico* analysis of microarray transcriptomic data generated from human CL lesions caused by *L*. *braziliensis* [12,13]. Expression of *IRF1*, *JAK2*, *IL6* and *IFI27* was significantly higher in CL lesions, corroborating our findings tiwht PBMCs (Fig 7A and 7B). Expression of *TLR2* and *IFIT1* was also significantly higher in CL lesions, suggesting that these molecules maybe differentially modulated at the infection site, compared to PBMCs. These findings indicate that differential expression we observed in PBMCs from both HPs and CL patients is also observed *in vivo*.

## Discussion

The initial encounter among *Leishmania* parasites and cells from the host´s immune system is fundamental in determining disease development or resistance. In naïve volunteers, this event is reflected in either high or low IFN-γ production [6]. Herein, we investigated the expression of a set of immune response-related genes that accompanies these polarized responses in PBMCs from naïve volunteers exposed to *L*. *braziliensis*. We initially confirmed earlier findings by Pompeu et al. [6] regarding two patterns of IFN-γ production in the peripheral response to *Leishmania*, suggesting that this dichotomy may be a common feature following exposure of naïve cells to these parasites. Importantly, this dichomoty is stable as LPs remained low IFN-γ producers 40 days after the initial investigation [6].

In parallel to *IFNG*, genes such as *CXCL10*, *IFI27*, *IL6* and *LTA* were upregulated in both HPs and LPs, though to different extents. IFN-γ enhances the production of CXCL10 [22] and CXCL10 activates NK cells, further inducing the secretion of IFN-γ [23]. Schnorr et al. suggested that NK cells maybe a source of IFN-γ in the response to *Leishmania* and since we also detected *IFNG* mRNA and IFN-γ protein after 72h of *L*. *braziliensis* stimulation, we suggest that NK cells may play a role. Also, *IFI27* is highly induced by type I IFN and IFN-α stimulates IL-12 secretion, promoting IFN-γ production [24]. We speculate that IFI27, highly expressed in HPs, could have contributed towards the greater IFN-γ level observed in HPs. On the other hand, IL6 drives the differentiation of CD4^+^ Th2 cells by inducing early production of IL4 [25] and by interfering with SOCS1 phosphorylation[26]. In experimental Visceral Leishmaniasis, IL-6 deficient mice showed enhanced control of *L*. *donovani* infection [27], suggesting a possible deleterious role for IL6, which was expressed >4-fold in HPs, compared to LPs. Apart from the commonly upregulated genes, HPs and LPs equally suppressed the expression of *CD180*, *LY86* and *TLR5*: CD180 belongs to the TLR family of pathogen receptor and it is associated with MD-1 (LY86). MD-1 cooperates with CD180 and TLR4 in the recognition of LPS [28] whereas TLR5 is a receptor for bacterial flagellin. Downmodulation of such pathogen recognition receptors in both HPs and LPs, we speculate, may limit the initial inflammatory reaction, enabling the establishment of infection.

Upon examination of genes uniquely upregulated in HPs, the pro-inflammatory signature suggested earlier is further supported by expression of *CCL7* (a chemoattractant for macrophages during inflammation), *IL8*, *IL1A* and *IL1B*, all of which have been detected in CL lesions [29] and TNF, a cytokine extensively associated with the pathogenesis of CL [4,30–32] also present in high IFN-γ producers, as described by Pompeu et al. [6]. *PTGS2*, the inducible Prostaglandin-endoperoxide synthase/cyclooxygenase, is also expressed in CL lesions caused by *L*. *braziliensis* [33] and, again, was upregulated uniquely in HPs. The top immune pathways enriched in HPs were IL-17 related. PBMCs from CL patients produce elevated levels of IL-17 compared to healthy controls [34] and Th-17-related cytokines are overexpressed in lesions from mucosal leishmaniasis patients [35], indicating an association between IL-17 and pathogenesis in CL. TREM1 is selectively expressed on neutrophils, monocytes and macrophages and engagement of this receptor leads to a pro-inflammatory immune response [36]. This pathway triggers expression of IL1B and TNF [37], all of which were upregulated in HPs. The possible engagement of TREM preferentially in HPs seems to suggest that a stronger anti-*Leishmania* response in humans maybe implicated in tissue destruction, leading to lesion development. Infection of Trem1-deficient mice with *L*. *major* induced a milder inflammatory infiltrate and smaller lesions but the absence of TREM1 signaling did not impair parasite control [38] suggesting that the TREM1 pathway is associated with excessive inflammation rather than the capacity to control experimental infection.

In contrast to HPs, LPs expressed mainly IL9, interferon-related genes (*IFI44*, *IFIT1* and *IFITM2*) and receptors *IL2RA*, *IL3RA* and *TLR2*. IL-9 has been associated with the Th2 phenotype: susceptible BALB/c mice infected with *L*. *major* expressed *IL9* [39] and crossing of IL-9 transgenic mice to Th2 cytokine-deficient mice promoted Th2 cytokine production [40]. *IL3RA (CD123*) is expressed by plasmacytoid DCs (pDCs) which can activate NK cells (rev. in [41]) again suggesting the participation of these cells in the early response to *L*. *braziliensis* [16]. Several transcripts of the IL2 pathway are present in CL lesions caused by *L*. *braziliensis* and certain IL2RA gene polymorphisms were associated with a poor IFN-γ response and lower activation of regulatory Foxp3^+^ cells [13]; IL2RA was also upregulated in LPs though herein we did not probe for any of these polymorphisms. Among the top two pathways identified in the LP response were "Role of Pattern Recognition Receptors in Recognition of Bacteria and Viruses" and " Interferon Signaling", corroborating the upregulation in IFN-related genes such as *IFI44*, *IFIT1* and *IFITM2*.

Based on the premise that HPs displayed a more inflammatory signature, compared to LPs, we hypothesized that features of the HP response, identified in PBMCs *in vitro*, had an *in vivo* counterpart, in CL patients. On the contrary, the *in vivo* equivalent of LPs would be SC individuals, characterized by the presence of lower IFN-γ and TNF production upon PBMC stimulation and absence of CL lesions [9]. A group of six genes differentially expressed in HPs and LPs allowed the discrimination of CL patients from SC individuals, suggesting that features of the early *in vitro* response to *L*. *braziliensis* are at play *in vivo*. As shown elsewhere, IFN-γ and CXCL10 levels are significantly higher in CL patients compared to SC individuals [16] which agrees with our data regarding HPs and LPs, respectively. We also observed upregulated expression of JAK2, IRF1, IL6 and IFI27 in CL lesions, strengthening our findings regarding the expression of these molecules in PBMCs from HPs and from CL patients.

In the present study we identified immune markers associated with High- and Low-IFN-γ producers, upon investigation of the peripheral response of naïve individuals to *L*. *braziliensis*. Certain markers were also expressed *in vivo*, in PBMCs from CL patients and SC individuals, respectively, and were further observed *in situ*, in CL lesions. Limitations of the study include the small number of individuals (n = 6) employed in the gene expression profiles and the limited number of genes selected for evaluation in CL patients and SC individuals. Despite these important limitations, our findings highlight the importance of addressing the initial response to *Leishmania* since, as shown here, such approaches can potentially lead to the identification of markers of CL development.

## Supporting Information

S1 Table Epidemiological parameters in HPs and LPs. (DOCX)

S2 Table Clinical and epidemiological parameters in CL patients and in individuals with subclinical infection. (DOCX)

S1 Fig Infection rate of PBMCs from High- and Low- IFN-γ producers. Macrophages obtained from HPs (n = 3) and LPs (n = 3) were infected with *L*. *braziliensis*. After 72h, glass coverslips were stained with hematoxylin-eosin and assessed with light microscopy for the percentage of infected macrophages (A) and the number of amastigotes per 100 macrophages (B) Data are from a representative experiment performed with HPs (n = 3) and LPs (n = 3) in quintuplicate. Data are shown as mean ± SEM.
(TIFF)

S2 Fig High- or Low- IFN-γ production is maintained upon stimulation with *Leishmania braziliensis* promastigotes. PBMCs were cultured with *L*. *braziliensis* for 72h. Supernatants were collected and levels of IFN-γ were determined by ELISA. Data from two experiments are shown individually for High Producers (HP) (*n* = 4) and Low Producers (LP) (*n* = 5).
(TIFF)
