# Supplementary material for: Gene Expression Profile of High IFN-γ Producers Stimulated with Leishmania braziliensis Identifies Genes Associated with Cutaneous Leishmaniasis
Source: PLoS Negl Trop Dis. 2016 Nov 21;10(11):e0005116. doi: 10.1371/journal.pntd.0005116 (PMC5117592; doi:10.1371/journal.pntd.0005116)
Supplement: S1 Table — (DOCX) [file pntd.0005116.s001.docx]

**Supplemental Table 1: Epidemiological parameters in HPs and LPs**

| Variable | HP (*n* =4) | LP (*n* = 5) | *p* value |
| --- | --- | --- | --- |
| Median age, years (range) | 29,5 (25 –35) | 26,8 (22 – 31) | 0.3079 |
| Male sex, No. (%) | 4 (100) | 2 (40) | 0.4071 |
