# Supplementary material for: Gene Expression Profile of High IFN-γ Producers Stimulated with Leishmania braziliensis Identifies Genes Associated with Cutaneous Leishmaniasis
Source: PLoS Negl Trop Dis. 2016 Nov 21;10(11):e0005116. doi: 10.1371/journal.pntd.0005116 (PMC5117592; doi:10.1371/journal.pntd.0005116)
Supplement: S2 Table — (DOCX) [file pntd.0005116.s002.docx]

**Supplemental Table 2: Clinical and epidemiological parameters in CL patients and in individuals with subclinical infection**

| Variable | Subclinical  infection (*n* =8) | Active CL  (*n* =5) | *p* Value |
| --- | --- | --- | --- |
| Median age, years (range) | 37 (21 –54) | 57 (49 – 66) | - |
| Male sex, No. (%) | 5 (62.5) | 1 (20.0) | 0.018 |
| Number of active lesions (mean) | - | 1,4 | - |
| Median DTH to leishmania, mm (range) | 10.6 (7-14) | 9.6 (6-12) | 0.488 |
